# Aluminum plasmonic photocatalysis

**DOI:** 10.1038/srep15288

**Published:** 2015-10-26

**Authors:** Qi Hao, Chenxi Wang, Hao Huang, Wan Li, Deyang Du, Di Han, Teng Qiu, Paul K. Chu

**Affiliations:** 1 Department of Physics and Jiangsu Key Laboratory for Advanced Metallic Materials, Southeast University, Nanjing 211189, P. R. China; 2 Department of Physics and Materials Science, City University of Hong Kong, Tat Chee Avenue, Kowloon, Hong Kong, P. R. China; 3 Southwestern Institute of Physics, Chengdu 610041, China

## Abstract

The effectiveness of photocatalytic processes is dictated largely by plasmonic materials with the capability to enhance light absorption as well as the energy conversion efficiency. Herein, we demonstrate how to improve the plasmonic photocatalytic properties of TiO_2_/Al nano-void arrays by overlapping the localized surface plasmon resonance (LSPR) modes with the TiO_2_ band gap. The plasmonic TiO_2_/Al arrays exhibit superior photocatalytic activity boasting an enhancement of 7.2 folds. The underlying mechanisms concerning the radiative energy transfer and interface energy transfer processes are discussed. Both processes occur at the TiO_2_/Al interface and their contributions to photocatalysis are evaluated. The results are important to the optimization of aluminum plasmonic materials in photocatalytic applications.

Plasmonic photocatalysis is a promising technique to boost the TiO_2_ photocatalytic ability to degrade organic compounds rapidly and non-selectively^1^. This technique utilizes localized surface plasmon resonance (LSPR) to form enhanced local electromagnetic fields around the TiO_2_ photocatalyst to improve the photocatalytic optical trapping capability and photoelectric conversion rate. The key is to fabricate precisely controlled nanostructures so that the LSPR spectrum is sufficiently coupled with the incident light^2^. In this case, plasmonics enhances light absorption and extends TiO_2_ absorption to a broad band^3^,^4^. However, utilization of light energy is still low when there is no overlap between the LSPR energy and TiO_2_ band gap^5^,^6^. Consequently, the photoelectric conversion rate is inadequate for plasmonic photocatalytic processes involving recalcitrant organic compounds such as pesticides and azo dyes. One solution to enhance the quantum efficiency and subsequently improve the photoelectric conversation is to couple the light with the LSPR energy at which TiO_2_ absorbs UV light. However, common plasmonic materials such as silver and gold have plasmonic bands in the visible range and extension of the LSPR response into the UV region is difficult due to the intrinsic limitations of the noble metals^7^,^8^,^9^.

Aluminum is an alternative plasmonic material with an extended response into the deep UV region^10^,^11^,^12^,^13^,^14^. The extended response in conjunction with the low cost and convenient manufacturing makes aluminum promising in UV plasmonics applications such as surface-enhanced fluorescence^15^,^16^, surface-enhanced Raman scattering^17^,^18^, and photovoltaics^19^. Although aluminum shows high-performance photocatalysis^20^, the applicability and associated mechanism have seldom been explored because of the high chemical reactivity of aluminum and difficulty in materials preparation^21^. The mechanisms responsible for general semiconductor/metal photocatalysis can be classified into two main categories: (1) LSPR-based electron-hole separation, namely radiative energy transfer, requiring the plasmonic band to be coupled with the TiO_2_ band gap and (2) Photo-induced hot electron transfer to a nearby reactant, namely interfacial charge transfer^22^. In this paper, these two processes are demonstrated to occur at the TiO_2_/Al interface and the underlying mechanism is discussed.

## Results and Discussion

**Figure 1:**
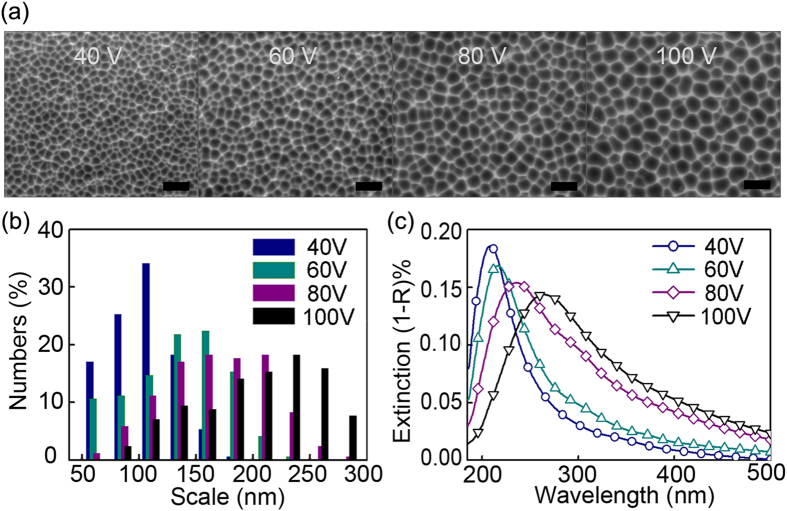
(**a**) SEM images of the aluminum foils with nano-void arrays at 40 V, 60 V, 80 V and 100 V with the scale bar being 500 nm. (**b**) Scale distribution of aluminum nano-voids. (**c**) UV-visible spectra of the aluminum foils.

A series of aluminum nano-void arrays with different diameters is prepared using the porous anodic alumina template to identify the scale suitable for plasmonic photocatalysis. The scanning electron microscopy (SEM) images acquired from the evolving nano-void structure on the aluminum foil are depicted in Fig. 1(a) at different voltages of 40 V, 60 V, 80 V, and 100 V. The metallic nano-void arrays possess several classes modes and are capable of facilitating strong coupling of incident light into LSPR modes, providing broad spectral tuning of the modes as well as offering large field enhancement^23^,^24^. The ranges of the nano-void structures are shown in Fig. 1(b) and the average diameters are 126 nm, 150 nm, 179 nm, and 217 nm at 40 V, 60 V, 80 V, and 100 V, respectively. The domain scale of the aluminum nano-voids becomes larger and the scale distribution becomes less uniform when the voltage is increased. The UV-visible spectra of the aluminum foils reveal absorption bands between 200 and 500 nm representing the surface plasmon resonance energy as shown in Fig. 1(c). The intense absorption is associated with the considerable enhancement in the electromagnetic field in the vicinity of the aluminum nano-voids. A reasonably strong interband transition in Al is localized at ~800 nm^6^ and the interband activity is weak below or above this energy. So it is reasonable to expect that Al nanoparticles support long-lived LSPR modes with high optical cross-sections and that are tunable over a wide energy range, deep into the UV^5^. Figure 1(c) confirms it by showing the LSPR absorption band of the aluminum void red-shifts with domain size scale and the coupling efficiency with TiO_2_ (3.2 eV) improves subsequently. When the LSPR spectra of the plasmonic materials shift closer to the interband transition, they broaden leading to a better coupling efficiency with the incident light. This enhanced field can be utilized to improve the photocatalytic performance by increasing light absorption, improving electron-hole generation, and heating the surroundings^25^.

**Figure 2:**
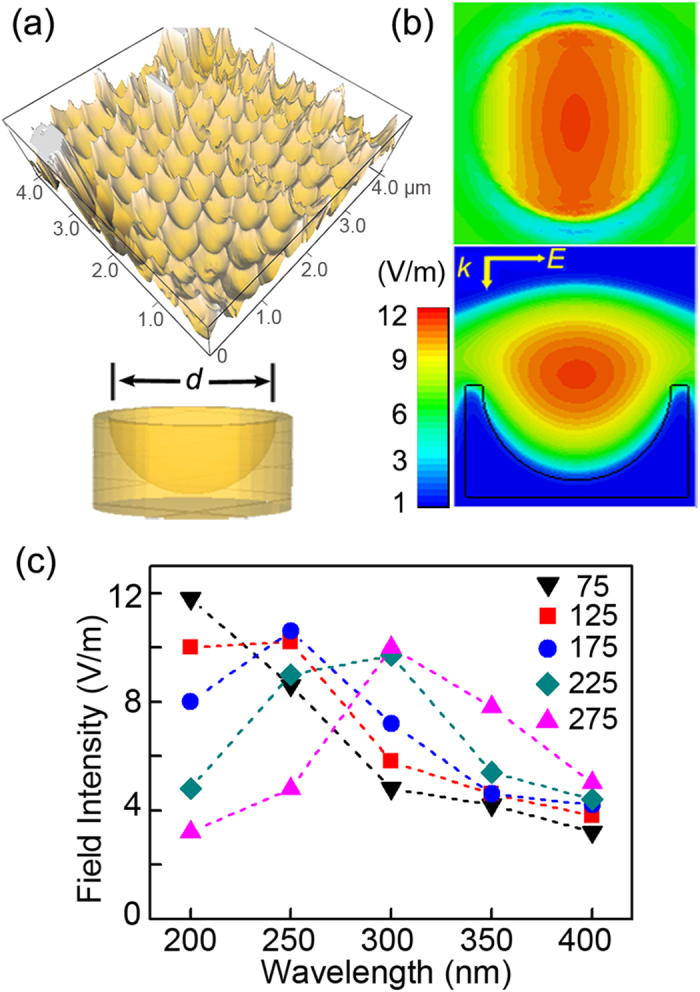
FDTD simulation results of the aluminum nano-void arrays. (**a**) Typical AFM image of the 100 V aluminum nano-void structure and three-dimensional model of the nano-void structure with *d* being the diameter of the hemisphere. (**b**) Cross-sectional and planar views of the calculated radial EM field components of the 100 V aluminum nano-void structure (*d* = 217 nm); the inset in (**b**) shows the *k*-vector and polarization of the incident light. (**c**) Calculated local electromagnetic field intensity of the nano-void arrays at different wavelengths for *d* = 75 nm, 125 nm, 175 nm, 225 nm, and 275 nm, respectively.

The aluminum LSPR spectra bear a strong relationship with the three-dimensional structure of the nano-voids. The finite-difference time-domain (FDTD) method is applied to simulate the setup to assess the field enhancement confined in the aluminum nano-voids, as shown in Fig. 2. Figure 2(a) presents a representative atomic force microscopy (AFM) image of the aluminum nano-void structure and three-dimensional aluminum nano-void model based on AFM (Figure S1) for the FDTD simulation. The cross-sectional and planar view of the calculated radial electromagnetic field components of the nano-void structure is displayed in Fig. 2(b). The incident radiation excites a plasmon trapped in the nano-void to create a large electromagnetic field and an amplitude enhancement can be observed from the aluminum nano-void model. The electromagnetic field are close to the central z-axis of the void and in less contact with the metal surface as confirmed by Mie theory^23^. When the plasmonic structure is coated with a TiO_2_ photocatalytic layer, the enhanced field is confined to the interior of the TiO_2_ layer and the plasmon-induced electron-hole pairs diffuse to the photocatalytic surface contributing to the photocatalytic reaction. The calculated local electromagnetic field intensity of the nano-void structure with different diameters *d* is monitored at different wavelengths to reveal the aluminum plasmonic band and the results are displayed in Fig. 2(c). It can be concluded that the plasmonic response in the near UV spectrum area increases with size thus promoting radiative energy transfer. It should be noted that in the experiment the nano-void are embedded in the metal films and there are coupling between the nano-voids. Both may produce additional plasmonic modes that selectively couple to the nano-void plasmons to produce bonding and anti-bonding hybridized states.

**Figure 3:**
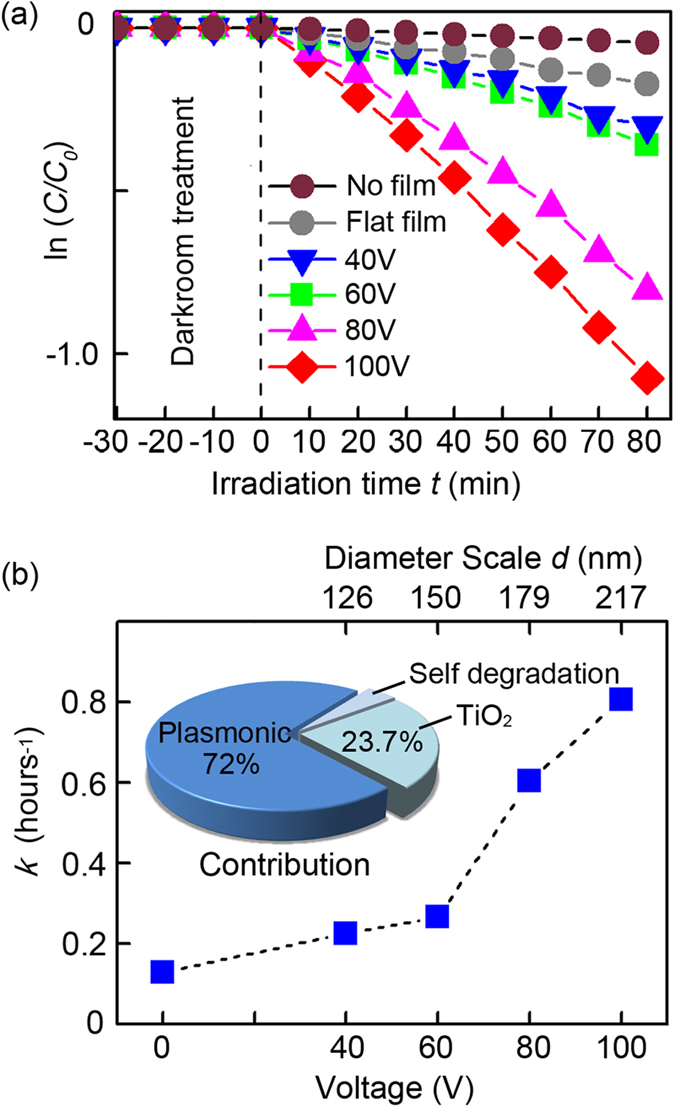
Photocatalysis data and analysis. (**a**) ln(C/C_0_) versus time curves under UV-visible irradiation acquired from the TiO_2_/Al films at 40 V, 60 V, 80 V, 100 V and flat TiO_2_/Al film without nano-voids and with no film. (**b**) Corresponding photocatalytic rate versus the TiO_2_/Al film fabrication voltage/nano-void diameter scale.

The TiO_2_/Al photocatalyst films are prepared by spin coating anatase TiO_2_ on the surface of the aluminum plasmonic films and the extinction spectra are shown in Figure S2. Radiative energy transfer is the essential and distinctive mechanism in TiO_2_/metal plasmonic photocatalysis. In this process, the coupling between the incident light and plasmonic materials forms an enhanced electromagnetic field which transfers the radiative energy from the plasmonic materials to the semiconducting TiO_2_^1^,^26^. The radiative energy boosts the photocatalysis efficiency when the LSPR energy is coupled with the TiO_2_ bandgap, otherwise the energy is more likely to decay non-radiatively through electron–phonon interactions and no significant enhancement in the photocatalytic activity can be observed^27^. To identify the importance of the coupling in plasmonic photocatalysis and elucidate the mechanism, photo-degradation experiments involving Rhodamine B are performed under UV−visible light illumination, as shown in Fig. 3. The change in the intensity of the Rhodamine B absorption peak at *λ* = 554 nm is used to determine the Rhodamine B concentration and specific information about the UV-visible spectra evolution is available in Figure S3. Besides, the TiO_2_/Al photocatalyst films show good repeatability and stability as illustrated in Figure S4.

The photocatalytic reaction follows the Langmuir–Hinshelwood adsorption model^28^, which can be simplified to the following pseudo-first-order expression: ln(*C/C*_*0*_) = -*kt*, where *C*_*0*_ and *C* represent the concentrations before and after irradiation, respectively. The photocatalytic rate *k* is the linear plot of ln(*C/C*_*0*_) versus irradiation time *t*. Figure 3(a) shows that the photocatalytic degradation rate *k* of Rhodamine B is significantly increased on the plasmonic TiO_2_/Al films compared to the flat TiO_2_/Al film. The calculated photocatalytic rate *k* is shown in Fig. 3(b), *k*_0_ = 0.035 h^−1^ for Rhodamine B self-degradation in water, *k*_*f*_ = 0.129 h^−1^ for flat TiO_2_/Al film without nano-void structures, and *k*_40_ = 0.226 h^−1^, *k*_*60*_* = *0.267 h^−1^, *k*_80_ = 0.605 h^−1^, and *k*_100_ = 0.806 h^−1^ for the plasmoninc TiO_2_/Al films at 40 V, 60 V, 80 V and 100 V, respectively. The photocatalysis efficiency is improved at higher voltages and this is because the hole-scale *d* of the “higher voltage” film is enlarged so that the coupling efficiency between the plasmonic spectrum and TiO_2_ bandgap is consequently improved (see Fig. 1c).

We take *k*_100_ as one unit and the calculated contributions for photocatalysis are plotted in the inset in Fig. 3(b). The enhancement originates from the combined LSPR effect and enlarged surface area. As shown in Fig. 3(a), the photocatalysis efficiency decreasing rate drops when *d* decreases and there is only a small change between *k*_40_ and *k*_*60*_. The plasmonic effect is expected to be negligible if *d* deceases continuously and we may simply consider that there is no plasmonic effect in the 40 V TiO_2_/Al film. Therefore, the contribution of the LSPR effect is 72.0% (*k*_100_ − *k*_40_). TiO_2_ photocatalysis accounts for 23.7% (*k*_40_ − *k*_0_) and Rhodamine B self-degradation *k*_0_ is 4.3%. In addition to the LSPR effect, the porosity results in a larger surface area to enhance the photocatalytic activity. The nano-voids are assumed to have a perfect hemispherical structure so that the surface area is the same for TiO_2_/Al films at different voltages regardless of the hole-scale *d*. Yet, *k*_40_ is still higher than *k*_*f*_, the photocatalysis rate of flat TiO_2_/Al, and this increment may step from the larger surface area and other factors. Here, the enhancement in the 100 V TiO_2_/Al photocatalytic activity rate compared with the photocatalysis rate of flat TiO_2_ is (*k*_100_ − *k*_*f*_)/*(k*_*f*_ − *k*_0_) = 7.2.

**Figure 4:**
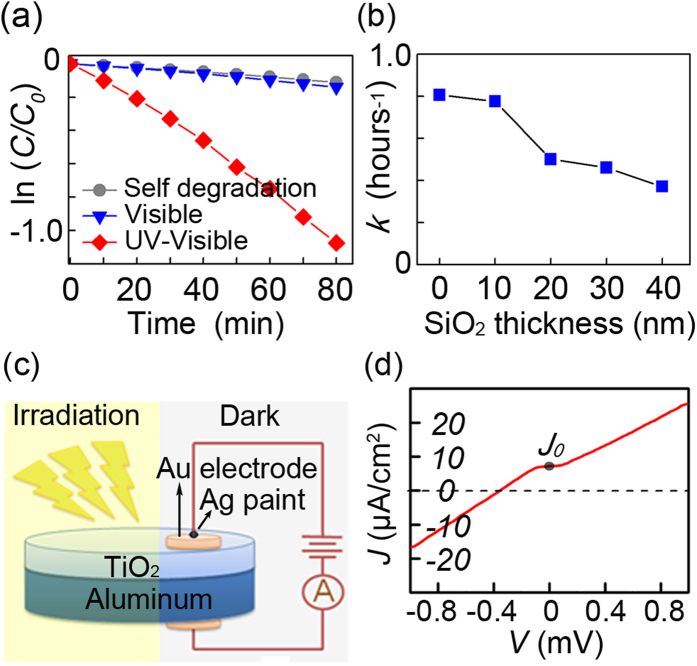
(**a**) ln(C/C_0_) versus time curves for 100 V TiO_2_/Al film under UV-visible and visible irradiation and with no film. (**b**) Photocatalytic rate for 100 V TiO_2_/Al film versus the SiO_2_ interlayer thickness under UV-visible irradiation. (**c**) Schematic of the photocurrent detection system. (**d**) The dependence of photocurrent density *J*-voltage *V* curves.

In-depth physical studies are performed on the TiO_2_/Al plasmonic photocatalysis system and the results are shown in Fig. 4. Figure 4(a) shows the degradation of Rhodamine B on the 100 V TiO_2_/Al films under UV-visible illumination as well as under visible illumination or UV-visible illumination with no films. There is no clear enhancement under visible light illumination suggesting that the LSPR field enhancement excited by UV irradiation is mainly responsible for the photocatalysis. The LSPR field enhancement can be classified into two subcategories: short range surface plasmon mode and long range surface plasmon mode, and both modes at the dielectric/metal interface can be used to enhance the photocatalysis performance. The short range mode is the key to the plasmonic nano-antennae and the short mode is typically located in the near-surface area (<20 nm) of the plasmonic materials and decays rapidly with distance^29^. Herein, the highest intensity hotspots are located in the center of the nano-void and this long range surface plasmon mode is caused by optical scattering which is not very sensitive to distance. Hence, the distance between the aluminum and TiO_2_ layers can be changed by adding a SiO_2_ layer with a predesigned thickness to adjust the field effect between the aluminum and TiO_2_. The addition of SiO_2_ may increase photocatalytic efficiency by cutting off the nonradiative energy transfer from TiO_2_ to aluminum and extending TiO_2_ absorption to the visible range. However, SiO_2_ may also block interfacial charge transfer process and decrease the photocatalysis efficiency by eliminating the Schottky barrier effect and decreasing the electron mobility. Figure 4(b) shows the degradation rate of Rhodamine B on the 100 V TiO_2_/SiO_2_/Al photocatalysis films. The decay rate *k* decreases slowly after 20 nm but the rate is higher than *k*_40_ (0.226 h^−1^, see Fig. 3a), indicating that the long range modes indeed still work. Furthermore, the decay rate *k* decreases rapidly when the SiO_2_ layer thickness is altered from 0 nm to 20 nm, indicating the possibility of other mechanisms at the TiO_2_/Al interface.

It is widely accepted that the interfacial charge transfer from TiO_2_ to metal plays an important role in plasmonic photocatalysis composite. At the TiO_2_/metal interface a significant redistribution of charge occurs depending on the metal work function and the semiconductor electron affinity. In the n-type TiO_2_ semiconductor, the charge redistribution forms the Schottky barrier which builds up an internal electric field inside the photocatalyst. The electric field facilitates the transfer of the photoexcited electrons from the semiconductor to the metal but prevents the opposite scenario, thereby suppressing electron/hole recombination and improving the quantum efficiency of photocatalysis. Unlike noble metal materials, aluminum is prone to oxidation and a 3 nm Al_2_O_3_ oxide layer is typically formed on the surface^30^. Oxidation may have a negative effect on the interfacial charge transfer due to the large work function of Al_2_O_3_. In this respect, the photocurrent of the 100 V TiO_2_/Al plamsonic film is monitored under UV-Vis irradiation in ambient air and the configurations are shown in Fig. 4(c). The electrode is kept in the darkness during the measurement to avoid interfering currents from the Au/TiO_2_ interface. Figure 4(c) shows photocurrent density *J* –voltage *V* dependences derived from the *I-V* curves with and without irradiation (see Figure S5). A strong photo-response occurs is revealed and the photocurrent increases linearly with voltages. The photocurrent density *J*_*0*_ of 7.2 μA/cm^2^ is obtained when the voltage is zero implying that the photo-induced electron/hole pairs of the TiO_2_ semiconductor are separated by an inner electric field and move to the semiconductor surface to promote photocatalysis. It can be deduced that the electric field is attributed to the built-in one arising from Schottky barrier effect. However, the 3 nm Al_2_O_3_ oxide layer on the surface impedes the formation of the Schottky barrier. Our results show that photocurrent still exist without external voltage, meaning that the native Al_2_O_3_ oxide layer cannot totally hamper the spontaneous charge transfer process at the TiO_2_/Al interface and this process may promote the photocatalysis degradation.

In summary, plasmonic aluminum nano-void arrays with tunable LSPR energies are fabricated to overlap the plasmonic spectrum and TiO_2_ bandgap. An enhanced decay rate of more than 7.2 folds is observed from the Rhodamine B degradation process. Although the coupling interaction between the aluminum and TiO_2_ is complicated, some basic mechanisms have been identified. First of all, LSPR improves photocatalysis efficiency *via* radiative energy transfer, especially when the aluminum plasmonic band is coupled with the TiO_2_ absorption band. Secondly, the interfacial charge transfer process contributes to photocatalysis at the TiO_2_/Al interface irrespective of the native Al_2_O_3_ layer on the surfaces. These results provide insights into aluminum plasmonic photocatalysis to improve photocatalysis efficiency.

## Methods

### Synthesis of TiO_2_/Al photocatalyst films

The aluminum films (99.99% pure, 15 × 5 × 0.2 mm) were degreased by acetone and electro-polished in a mixture of ethanol and perchloric acid with a volume ratio of 5:1 at a constant direct-current voltage of 15 V for 3 min to remove surface impurities. The polished aluminum films were rinsed in distilled water and anodized separately in a phosphoric acid solution (0.5 m) at a constant direct-current voltage of 100 V (80 V, 60 V, and 40 V) at 10 °C for 2 h followed by immersion in a mixture of chromic acid (1.8 weight %) and phosphoric acid (6 wt %) at 75 °C (1:1 in volume). The obtained aluminum plasmonic nano-void structures are shown in Fig. 1(a). The TiO_2_/Al photocatalyst films were prepared by spin coating anatase TiO_2_ (2 g in 60 ml water) on the surface of obtained aluminum films at 1,800 rpm for 30 s (Laurell spin coater model WS-400BZ-6NPP-Lit). The films were baked at 120 °C for 5 min and then annealed at 300 °C for 2 h. A TiO_2_ film with a thickness of 90 nm was formed. Deposiition of the Au electrode and SiO_2_ was performed on a physical vapor deposition system with a film monitor to control the film thickness (Taiyao FTM-V).

### Photocatalysis

The concentration of the Rhodamine B solution was 10 ppm and the volume of the solution was 20 mL. The photo-degradation experiments were performed on the photocatalyst films under UV−visible and visible light illumination (200 W Xe lamp without and with UV filter to block UV light < 400 nm). The distance between the light source and photocatalyst films was 20 cm. The solution was kept in darkness for 30 minutes prior to light irradiation to exclude the influence from molecule absorption on the container. During illumination, distilled water was added continuously to the solution to keep the volume constant.

### FDTD simulation

The Drude model was adopted for the FDTD simulation and the optical parameters were obtained from the reference^31^. The domain size was set to be (*d* + 20, *d* + 20, 1000) nm, where *d* represented the scale size of the nano-void. A 200 nm thick perfect matched layer was set at the top and bottom of the domain area, respectively. The tetrahedral mesh method was adopted and the minimum/maximum edge length was 10.95/42.2 nm. The number of total mesh tetrahedrons was about 200,000. The modes were excited by a broad band plane wave source between 200 nm and 2,000 nm perpendicular to the plane of the structure and the incident light intensity was 1 V/m. The FDTD simulation was performed using a commercial CST software.

### Instrumentation and data acquisition

The anatase TiO_2_ nanoparticles T104949 were purchased from Aladdin and Image-Pro Plus version 6.0 was used to conduct the statistical analysis of the nano-void scale. The UV-visible absorption spectra were acquired from the TiO_2_/Al photocatalyst films on a PerkinElmer LAMBDA750 spectrophotometer. Rhodamine B was purchased from Sigma-Aldrich. The absorbance of Rhodamine B solution was measured with an UV−visible spectrophotometry (HITACHI U-3900) every ten minutes and irradiation ceased during spectrum acquisition.

## Additional Information

**How to cite this article**: Hao, Q. *et al.* Aluminum plasmonic photocatalysis. *Sci. Rep.* **5**, 15288; doi: 10.1038/srep15288 (2015).

## Supplementary Material

Supplementary Information
