# Supplementary material for: Aluminum plasmonic photocatalysis
Source: Sci Rep. 2015 Oct 26;5:15288. doi: 10.1038/srep15288 (PMC4620498; doi:10.1038/srep15288)
Supplement: Supplementary Information [file srep15288-s1.doc]

Supporting Information

Aluminum plasmonic photocatalysis

Qi Hao,^1,2^ Chenxi Wang,^2,3^ Hao Huang,^1^ Wan Li,^2^ Deyang Du,^1^ Di Han,^1^ Teng Qiu,^1^,^*^ & Paul K. Chu^2,*^

^1^Department of Physics and Jiangsu Key Laboratory for Advanced Metallic Materials, Southeast University, Nanjing 211189, P. R. China, ^2^Department of Physics and Materials Science, City University of Hong Kong, Tat Chee Avenue, Kowloon, Hong Kong, P. R. China, ^3^Southwestern Institute of Physics, Chengdu 610041, China


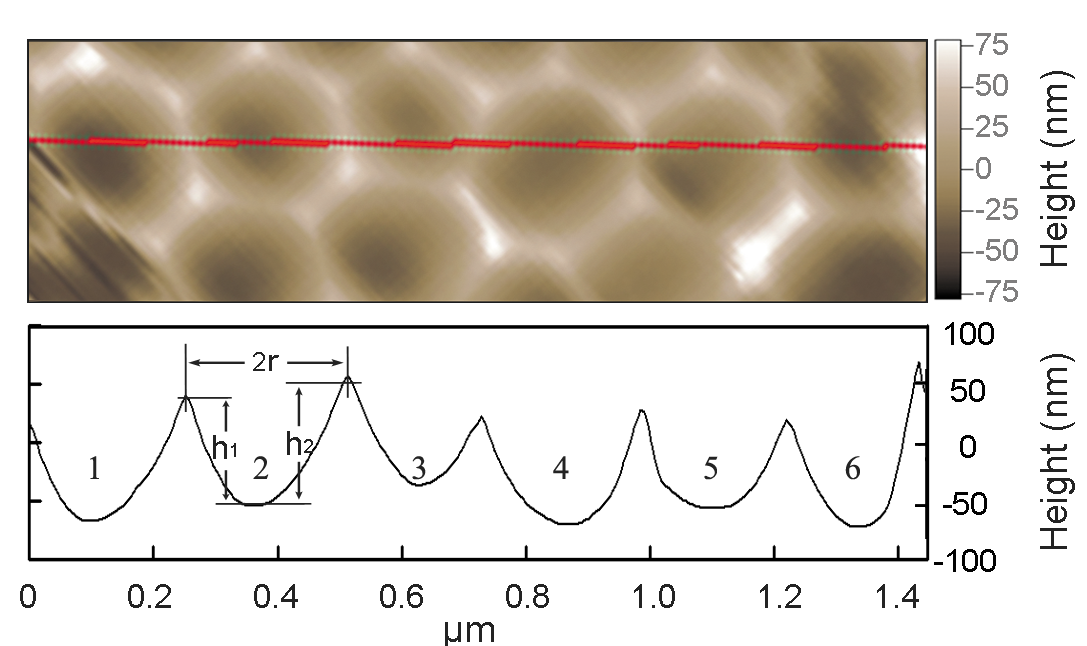


|  | 1 | 2 | 3 | 4 | 5 | 6 |
| --- | --- | --- | --- | --- | --- | --- |
| r (nm) | 126.0 | 131.7 | 106.5 | 128.8 | 117.7 | 106.4 |
| (h1+h2)/2  (nm) | 118.3 | 127.5 | 94.8 | 118.7 | 99.9 | 145.2 |

**Figure S1.** Typical AFM image of the 100 V aluminum film. The curve shows the depth of the red line in the AFM figure, where *r* represents the radius, *h_1_* represents left side height of the nano-void structure and *h_2_* represents the height of the right side. The data indicate that the aluminum nano-void structure is almost hemispherical in shape.


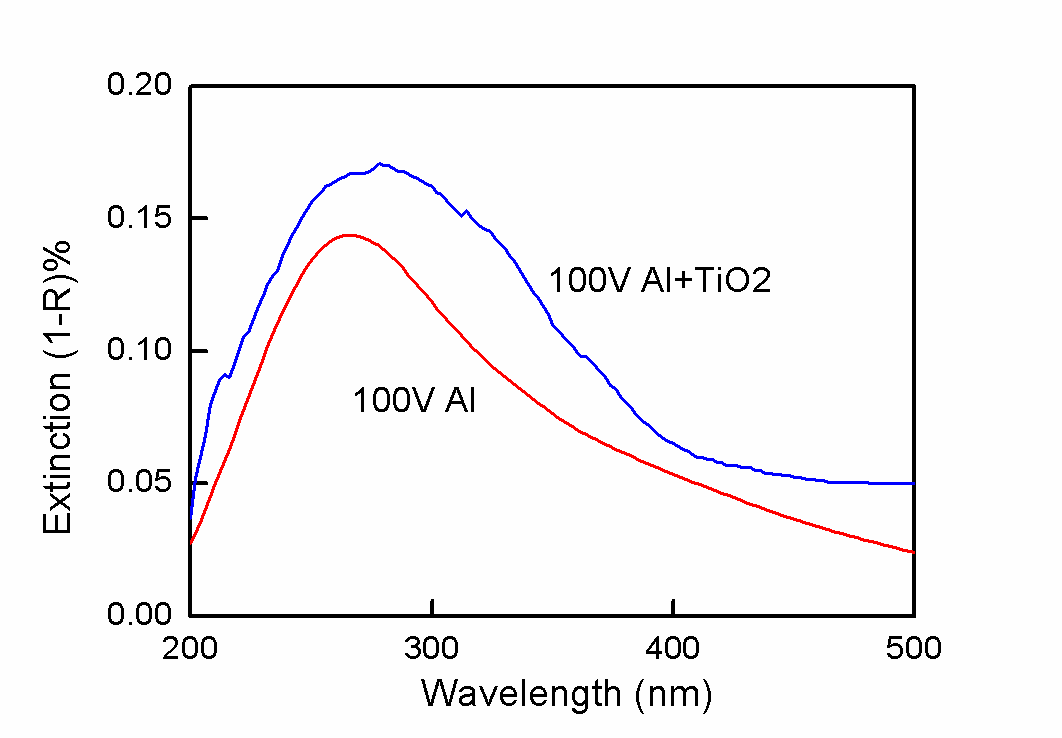


**Figure S2.** UV-vis spectra of the 100V aluminum film with and without TiO_2_ coating.


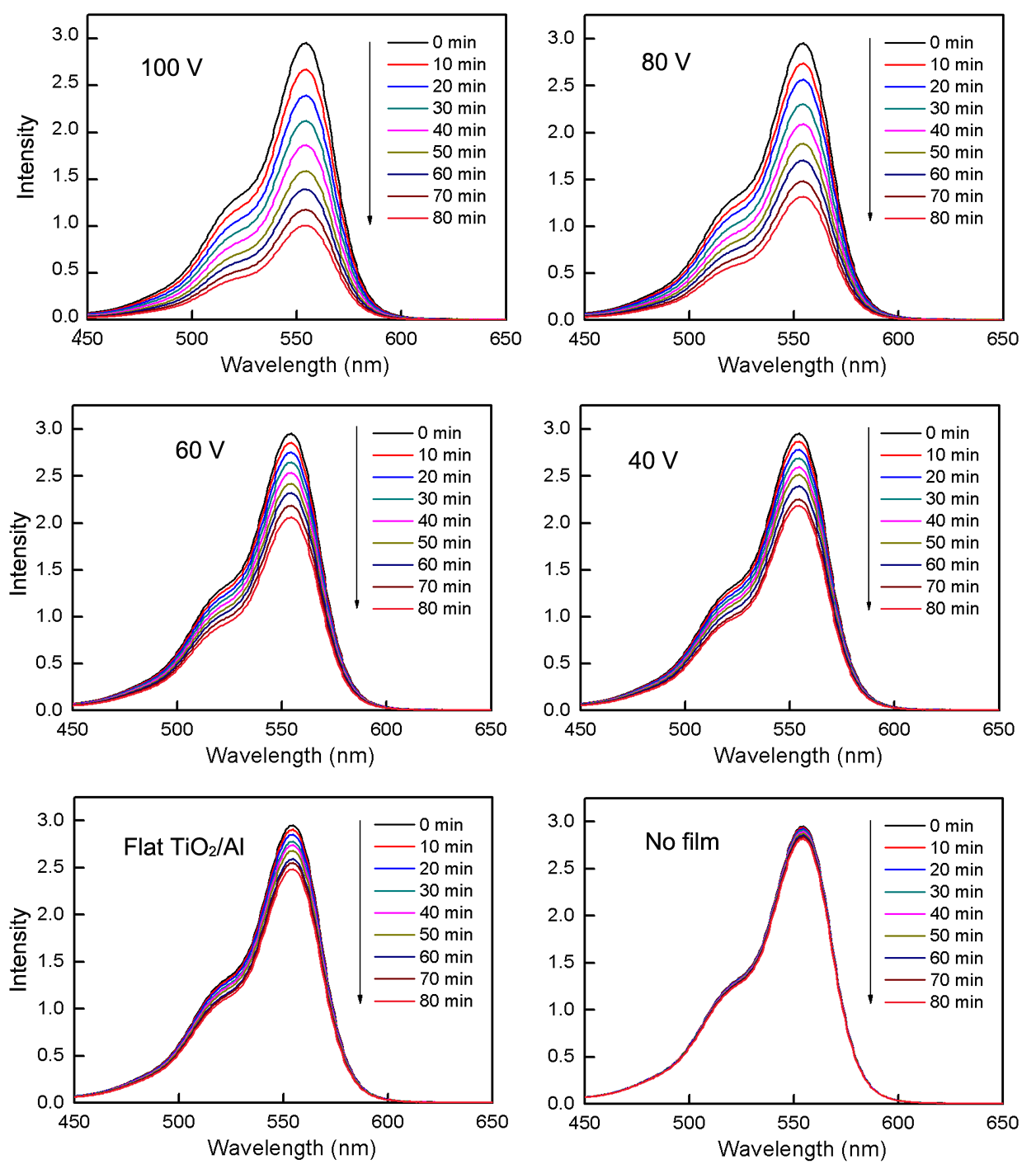


**Figure S3.** UV-vis spectra evolution of Rhodamine B solution under UV-Vis irradiation with 40 V, 60 V, 80 V, 100 V TiO_2_/Al film, flat TiO_2_/Al film and no film.


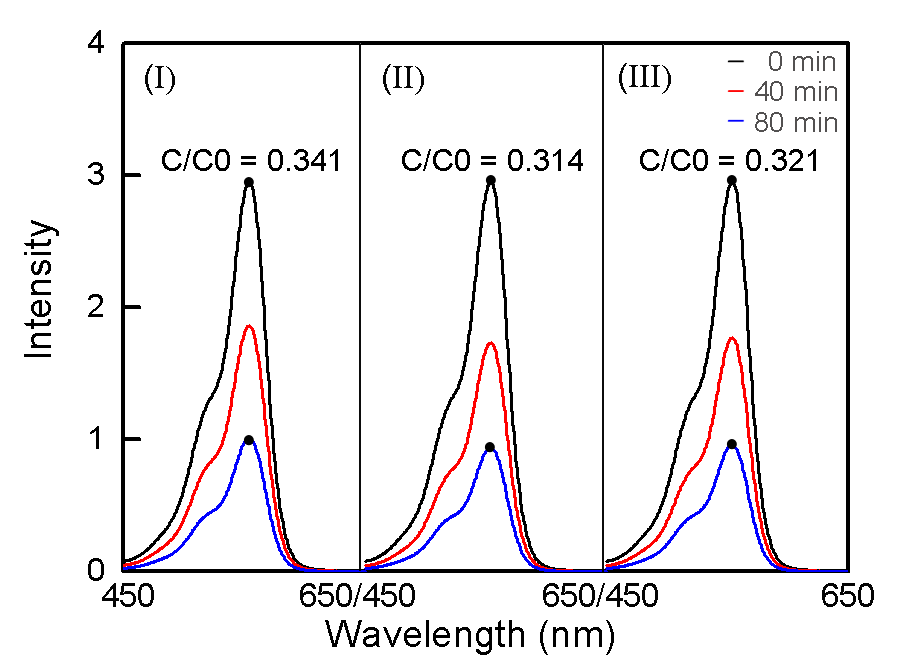


**Figure S4.** Repeatability and stability of the aluminum plasmonic photocatalyst. The photocatalysis experiment is conducted continuously with a 100 V TiO_2_/Al film three times under UV-vis illumination and the UV-vis spectra evolution of Rhodamine B solution is shown. *C*/*C*_0_ = 0.341, 0.314 and 0.321 from left to right, where *C* and *C_0_* represent the concentrations before and after irradiation for 80 min, respectively. The results show that the stability of the photocatalyst film is good because of the 3 nm thick Al_2_O_3_ oxide layer on the surface protecting the aluminum from further oxidation.


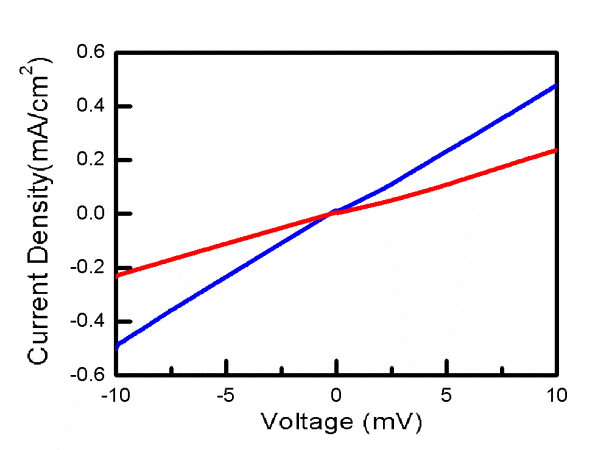


**Figure S5.** Comparsion of the current density-voltage characterisics of the 100 V TiO_2_/Al photocatalyst films device with (blue) and without irradiation (red).
